# Percutaneous evacuation of diffuse pulmonary interstitial emphysema by lung puncture in a baby with extremely low birth weight: a case report

**DOI:** 10.1186/1752-1947-6-325

**Published:** 2012-09-26

**Authors:** Masahiro Watanabe, Nobuo Momoi, Maki Sato, Hayato Go, Takashi Imamura, Masatoshi Kaneko, Mitsuaki Hosoya

**Affiliations:** 1 Department of Pediatrics, Fukushima Medical University, 1st Hikarigaoka, Fukushima, 960-1295, Japan

**Keywords:** Lung puncture, Pulmonary interstitial emphysema, Baby with extremely low birth weight

## Abstract

**Introduction:**

Pulmonary interstitial emphysema is a serious complication of mechanical ventilation and can become life-threatening if progression occurs. Therapeutic lung puncture is a treatment option for severe pulmonary interstitial emphysema but has a limited use in babies with extremely low birth weight. We present a case of pulmonary interstitial emphysema in a Japanese baby (1-day-old) boy with extremely low birth weight. The emphysema was successfully decompressed by therapeutic lung puncture performed with a trocar catheter.

**Case presentation:**

The baby was born with a weight of 420g, which, to the best of our knowledge, is the lowest reported birth weight among babies with pulmonary interstitial emphysema. A chest X-ray on postnatal day 2 revealed pulmonary interstitial emphysema, which gradually progressed to diffuse pseudocystic changes. His condition became life-threatening despite the use of high-frequency oscillatory ventilation and lateral decubitus positioning. We evacuated the pulmonary interstitial emphysema by lung puncture with a trocar catheter to avoid respiratory and cardiovascular collapse. This resulted in adequate evacuation of the emphysema and a dramatic improvement in his clinical condition.

**Conclusions:**

Therapeutic lung puncture performed with a trocar catheter is beneficial in babies with extremely low birth weight and diffuse pulmonary interstitial emphysema. This treatment option may be broadly applicable, especially in an emergency situation.

## Introduction

Pulmonary interstitial emphysema (PIE) is a serious complication of mechanical ventilation, especially in extremely premature babies with respiratory distress syndrome (RDS). PIE causes respiratory deterioration and becomes a life-threatening condition if progression occurs. Therapeutic lung puncture has been described as a treatment for severe PIE. However, its use became limited in a few reported cases because of extremely low birth weight (ELBW) [1,2]. A treatment strategy for such cases has yet to be established. Here, we report a case of diffuse PIE decompressed by lung puncture performed with a trocar catheter in a baby with ELBW and a weight of 420g.

## Case presentation

A 1-day-old, 420g, Japanese boy was born through Cesarean section after a 25-week gestation to a 34-year-old, gravida 2, para 1 mother with pregnancy-induced hypertension. The Apgar scores of the baby were 4 and 8 at 1 and 5 minutes, respectively. He was placed on mechanical ventilation, and surfactant replacement therapy was administered for RDS. However, he developed pulmonary hemorrhage, and a chest X-ray revealed the deterioration of lung aeration. Surfactant replacement therapy was administered again, resulting in an improvement in oxygenation.

**Figure 1:**
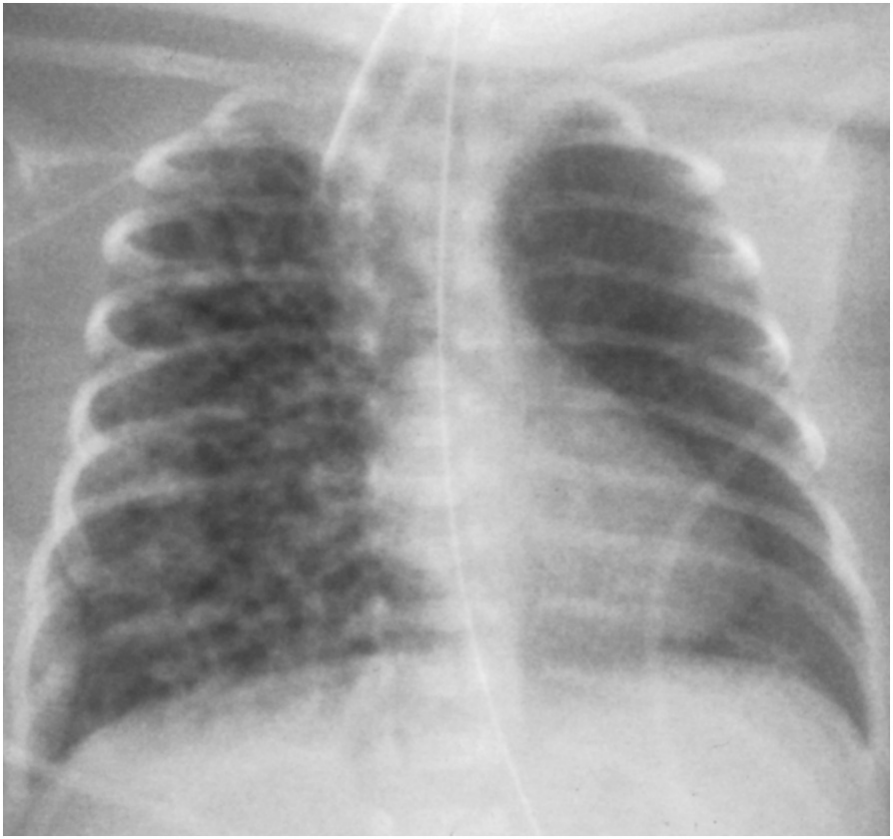
**A chest X-ray obtained on postnatal day 6.** The chest X-ray shows diffuse pulmonary interstitial emphysema over the entire right lung field.

**Figure 2:**
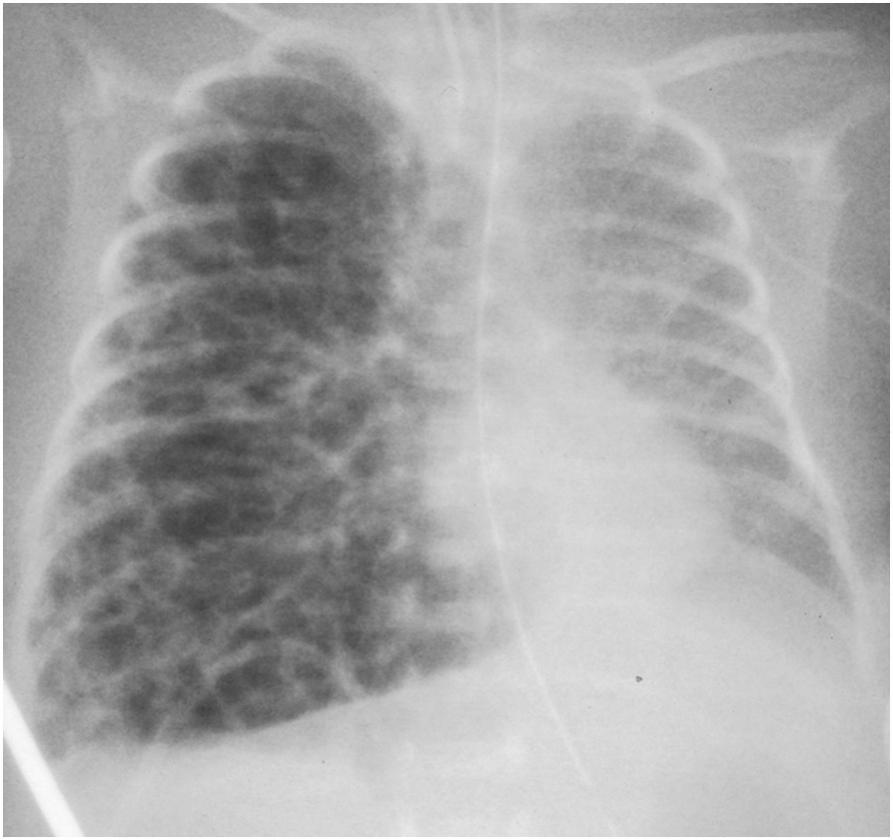
**A chest X-ray obtained on postnatal day 11.** Diffuse pulmonary interstitial emphysema with growing pseudocystic changes in the right lung field can be seen on this X-ray.

**Figure 3:**
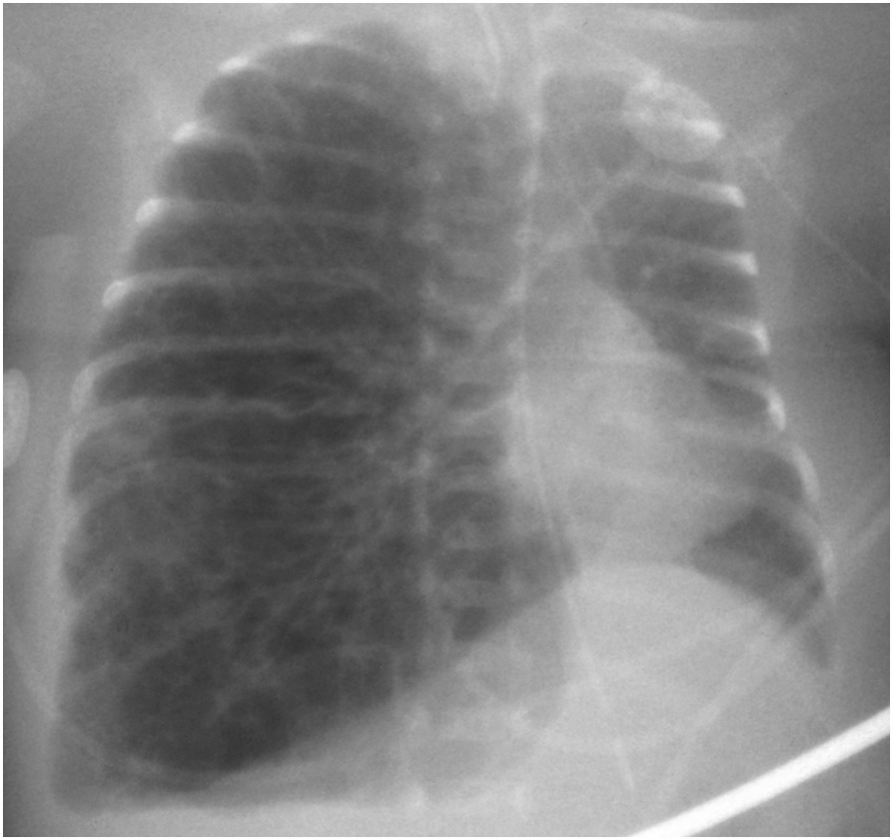
**A chest X-ray obtained before lung puncture.** The chest radiograph obtained just before decompression shows diffuse pulmonary interstitial emphysema in the right lung and a mediastinal shift to the left.

A chest X-ray obtained on postnatal day 2 revealed PIE in the right lung. High-frequency oscillatory (HFO) ventilation was initiated to prevent the progression of PIE. However, his condition gradually progressed over his entire right lung field (Figure 1). He was treated with right lateral decubitus positioning from day 8 and steroid therapy (hydrocortisone, 2mg/kg per day for 3 days) from day 10. However, the PIE worsened progressively and growing diffuse pseudocystic changes occured (Figure 2). Additional nitric oxide inhalation therapy was administered from day 13. Unfortunately, a sudden deterioration occurred at postnatal day 16. His systemic blood pressure (measured in millimeters of mercury) decreased from the 60s to the 40s, and his blood oxygen saturation at 100% fractional inspired oxygen concentration fell to 80%. A chest X-ray revealed progressively expanding PIE on the right side and a marked shift of the mediastinum to the left (Figure 3).

**Figure 4:**
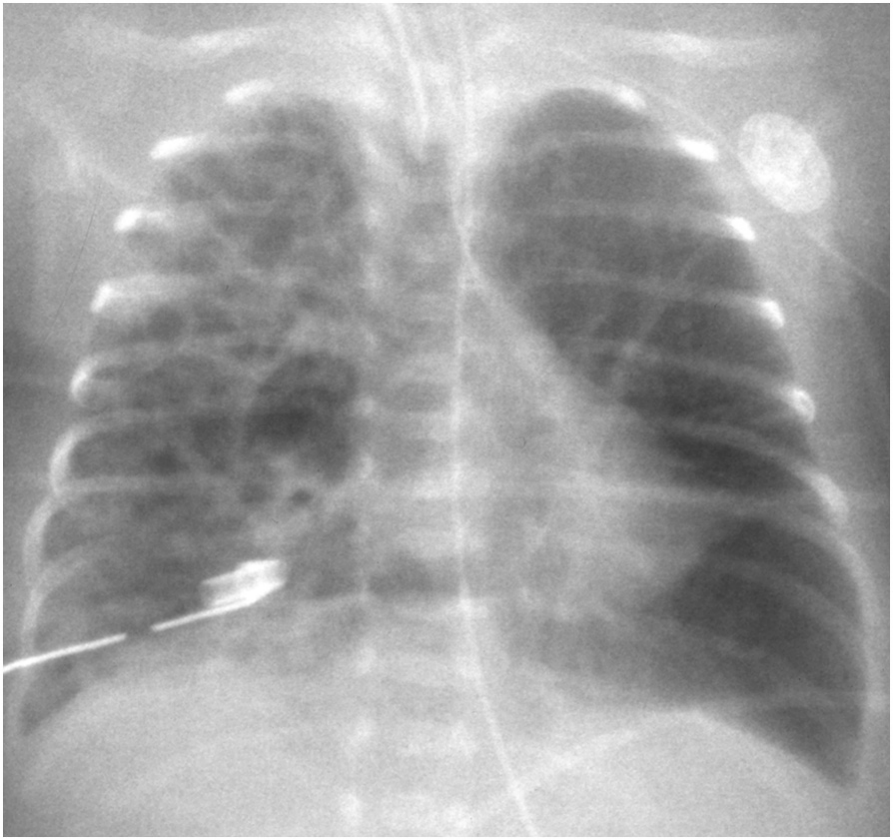
**A chest X-ray obtained after lung puncture.** Decompression of diffuse pulmonary interstitial emphysema was performed with a trocar catheter. The mediastinal shift improved after decompression.

**Figure 5:**
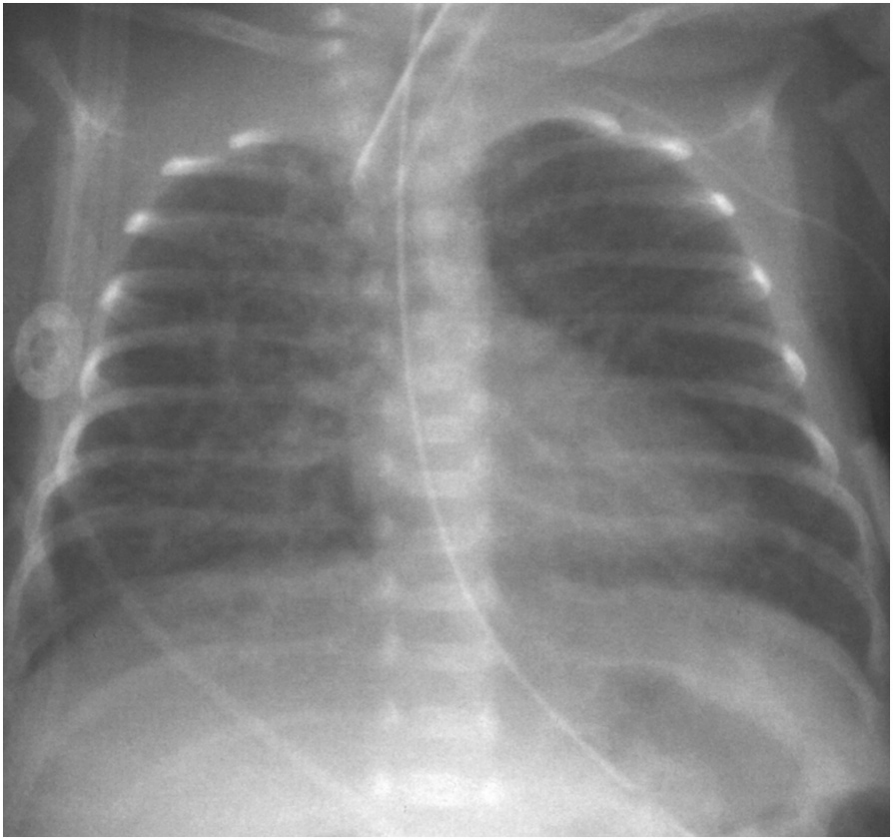
**A chest X-ray obtained on postnatal day 37.** Almost complete regression of pulmonary interstitial emphysema can be seen.

His clinical condition rapidly deteriorated to the same pathophysiological condition as that of tension pneumothorax. Therefore, we chose to evacuate the PIE by percutaneous lung puncture. A peripheral intravenous catheter (24 gauge; Terumo, Tokyo, Japan) was inserted into his right lower lung. However, evacuation with this catheter was insufficient, and an Argyle™ trocar catheter (10 French; Nippon Sherwood Medical Industries Ltd, Tokyo, Japan) was inserted into the same location. A total of 24cm of water was suctioned with the trocar catheter, resulting in adequate evacuation of the PIE, resolution of the mediastinal shift, and dramatic improvement in clinical condition (Figure 4). We used thoracic transillumination to monitor both the effectiveness of evacuation and the development of accidental pneumothorax. The drainage was discontinued 5 days after the lung puncture, and there was no PIE recurrence (Figure 5). After complete resolution of PIE, bilateral bubbling shadows gradually appeared on chest X-rays. Chronic lung disease was diagnosed, and systemic and inhaled steroids were administered. He was weaned from HFO ventilation on day 34 and from ventilatory support on day 68.

## Discussion

PIE is a serious complication of mechanical ventilation, especially in premature babies with RDS, but has become rare since the widespread use of prenatal maternal steroid administration and postnatal surfactant replacement therapy [3]. However, development of PIE may be a cause of respiratory deterioration, resulting in a life-threatening condition. Conservative treatments for PIE include HFO ventilation, lateral decubitus positioning [4], and systemic steroid administration [5]. For cases of PIE refractory to these treatments, selective bronchial intubation [6,7], balloon occlusion of the ipsilateral bronchus [8,9], and surgical resection of the affected lobes have been reported to be effective treatment strategies [10].

Therapeutic lung puncture has been described as a treatment for severe PIE but is not reported in many ELBW babies, for whom an effective strategy has yet to be established. Milligan *et al*. [2] described six babies (842 to 2400g), including two ELBW babies with life-threatening diffuse PIE, who were successfully treated by puncture of the affected lung and drainage of the resulting pneumothorax. Dördelmann *et al*. [1] reported an ELBW baby (765g) with PIE whose ipsilateral lung was punctured with a pigtail catheter to create and subsequently drain a pneumothorax. Our patient weighed 420g, which, to the best of our knowledge, is the lowest reported birth weight among ELBW babies with PIE. In this case, the PIE gradually progressed to diffuse pseudocystic changes and caused a life-threatening condition despite the use of HFO ventilation and right lateral decubitus positioning. We finally chose to evacuate the PIE by puncturing the lung percutaneously to avoid respiratory and cardiovascular collapse. We placed an Argyle™ trocar catheter into the pulmonary interstitial space to ensure the evacuation. Puncturing at just one location reduced not only the pseudocyst in the right lower lung but also the widespread ipsilateral PIE. This evacuation appears to indicate both the usefulness and safety of lung puncture. It has been reported that the gas in pulmonary interstitial tissue pathologically accumulates within the lymphatic system of the lung in preterm babies [11] and that air-filled cysts are frequently interconnected in the immature lung [12]. A single-site lung puncture will probably succeed even in diffuse PIE cases because of these pathological characteristics. On the other hand, the risks of airway injury and air leakage are considered to be negligible because small airways, including the alveolar air space, are collapsed as a result of excessively increased gas pressure in the pulmonary interstitial tissue. PIE with a mediastinal shift toward the contralateral side is thought to present the same pathophysiological condition as that of tension pneumothorax. This condition leads to worsening hypoxia and compromised venous return. Therefore, aggressive management with catheter drainage of the PIE should be stressed.

## Conclusions

Therapeutic lung puncture with a trocar catheter may be a useful treatment option for diffuse PIE, even in babies with ELBW. This treatment option may be broadly applicable, especially in an emergency medical condition.

## Consent

Written informed consent for publication of this case report and any accompanying images was obtained from the patient’s parents. A copy of the written informed consent is available for review by the Editor-in-Chief of this journal.

## Abbreviations

ELBW: extremely low birth weight; HFO: high-frequency oscillatory (ventilation); PIE: pulmonary interstitial emphysema; RDS: respiratory distress syndrome.

## Competing interests

The authors declare that they have no competing interests.

## Authors’ contributions

MW wrote the first draft of the manuscript. MS, HG, TI, and MK were the patient’s attending doctors and collected the relevant data. NM and MH revised the manuscript and contributed to its concept. All authors read and approved the final manuscript.
